# [15]aneN_4_S: Synthesis, Thermodynamic Studies and Potential Applications in Chelation Therapy

**DOI:** 10.3390/molecules19010550

**Published:** 2014-01-03

**Authors:** Nuno Torres, Sandrina Gonçalves, Ana S. Fernandes, J. Franco Machado, Maria J. Villa de Brito, Nuno G. Oliveira, Matilde Castro, Judite Costa, Maria F. Cabral

**Affiliations:** 1 Instituto de Investigação do Medicamento (iMed.ULisboa), Faculdade de Farmácia, Universidade de Lisboa, Av. Prof. Gama Pinto, Lisboa 1649-003, Portugal; E-Mails: nunotorr@gmail.com (N.T.); sandrinagoncalves@campus.ul.pt (S.G.); joaomachado@campus.ul.pt (J.F.M.); ngoliveira@ff.ul.pt (N.G.O.); mcastro@ff.ul.pt (M.C.); jcosta@ff.ul.pt (J.C.); 2 CBIOS, Universidade Lusófona Research Center in Biosciences & Health Technologies, Campo Grande 376, Lisboa 1749-024, Portugal; E-Mail: ana.fernandes@ulusofona.pt; 3 CQE, Instituto Superior Técnico, Universidade de Lisboa, Av. Rovisco Pais, Lisboa 1049-001, Portugal; E-Mail: mjbrito@fc.ul.pt; 4 DQB, Faculdade de Ciências, Universidade de Lisboa, Campo Grande, Lisboa 1749-001, Portugal

**Keywords:** macrocyclic compounds, thiatetraaza, stability constants, spectroscopic studies, chelation therapy, mercury(II) chelator, copper(II) chelator

## Abstract

The purpose of this work was to synthesize and characterize the thiatetraaza macrocycle 1-thia-4,7,10,13-tetraazacyclopentadecane ([15]aneN_4_S). Its acid-base behaviour was studied by potentiometry at 25 °C and ionic strength 0.10 M in KNO_3_. The protonation sequence of this ligand was investigated by ^1^H-NMR titration that also allowed the determination of protonation constants in D_2_O. Binding studies of [15]aneN_4_S with Mn^2+^, Fe^2+^, Co^2+^, Ni^2+^, Cu^2+^, Zn^2+^, Cd^2+^, Hg^2+^ and Pb^2+^ metal ions were further performed under the same experimental conditions. The results demonstrated that this compound has a higher selectivity and thermodynamic stability for Hg^2+^ and Cu^2+^, followed by Ni^2+^. The UV-visible-near IR spectroscopies and magnetic moment data for the Co(II) and Ni(II) complexes indicated a tetragonal distorted coordination geometry for both metal centres. The value of magnetic moment and the X-band EPR spectra of the Cu(II) complex are consistent with a distorted square pyramidal geometry.

## 1. Introduction

Exposure to toxic metals is associated with the potential development of numerous health effects, including different types of cancer [1,2]. Humans are exposed to metals and their compounds through environmental and occupational scenarios and also by food consumption, being the adverse effects dependent on dose, period of exposure and metal bioavailability [3]. In fact, metals can disturb the normal function of several organ systems and generate different toxicological effects through their excess, lack or imbalance [4]. The mainstay treatment against metal toxicity is chelation therapy [5,6], where appropriate chelators are used to remove metals from the organism [7], and thereby reduce toxicity [8,9]. However, despite the available chelators, the search for new effective chelating compounds is still needed, since the ones currently used in clinic present a number of toxic side effects, low metal specificity and controversial efficacy [5,9,10].

Previously, we have explored the possible use of macrocyclic compounds for medical applications, namely in chelation therapy [7,11]. These compounds may exhibit important properties such as less toxicity, high kinetic and thermodynamic stabilities [12], rendering them very promising agents in this context. Macrocycles are also advantageous in terms of selectivity, since they have more rigid structures and can thus inflict specific coordination geometry to the metal ion, while open chain chelators can adapt more easily to the geometric requirements of the metal centre [7,13]. 

**Figure 1:**
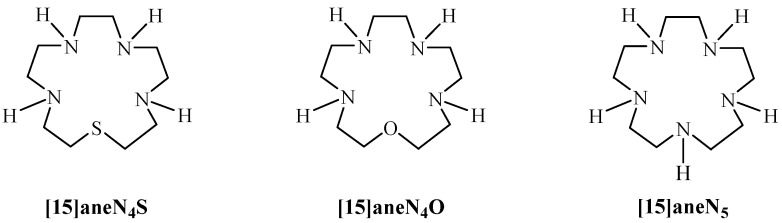
[15]aneN_4_S, [15]aneN_4_O and [15]aneN_5_ macrocycles.

A large amount of data has been published on macrocyclic ligands containing only nitrogen, only sulphur or both as donor atoms, namely tetraaza- [13], thiadiaza- [14], thiatriaza- [15], dithiadiaza- [16,17,18,19,20], dithiatriaza- [20] or pentathia- macrocycles [15]. However, there is scarce information on thiatetraaza compounds. In fact, only one thiatetraaza macrocycle, 1-thia-4,7,11,14-tetraazacyclohexadecane, was studied [21]. In the present work, we investigate a 15-membered thiatetraaza macrocyclic compound, 1-thia-4,7,10,13-tetraazacyclopentadecane ([15]aneN_4_S, Figure 1). In this context, we address the synthesis and characterization of [15]aneN_4_S and assess its potential as a chelating agent. To accomplish this aim, the acid-base behaviour of this macrocycle was studied and its ability to specifically coordinate with several divalent ions was evaluated. 

## 2. Results and Discussion

### 2.1. Synthesis and Characterization

**Scheme 1:**
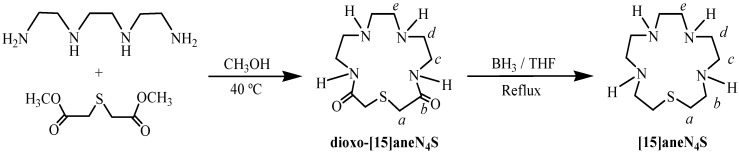
Schematic synthesis of [15]aneN_4_S.

The macrocycle 1-thia-4,7,10,13-tetraazacyclopentadecane ([15]aneN_4_S) was prepared according to the reactions depicted in Scheme 1. The first step involved the synthesis of the precursor diamide, 1-thia-4,7,10,13-tetraazacyclopentadecane-3,14-dione (dioxo-[15]aneN_4_S) by reaction of the dimethyl ester of thiodiglycolic acid, prepared according to a reported procedure [22], with triethylenetetramine in dry methanol at 40 °C, under N_2_ for nine days. The general procedure was similar to those reported in the literature by Tabushi *et al.* [23] and by Steenland *et al.* [24], who obtained this macrocycle with 15% to 17% yield. However, some modifications were introduced in the present work, such as controlled reaction temperature, dried solvent in a larger volume and nitrogen atmosphere, which led to the highly improved yield of 74% on dioxo-[15]aneN_4_S synthesis. The reduction of this cyclic diamide with borane, in refluxing dry THF under nitrogen for eight hours, afforded the [15]aneN_4_S. After purification by chromatography, the trihydrocloride salt was obtained by addition of 37% HCl until pH ≈ 2. A yield of 68% was obtained, which is within the range reported for similar compounds (60%–80%) [23].

The structure of the compound was confirmed by ^1^H and^ 13^C-NMR spectroscopy (Table S1 and Figures S1–S2 in Supplementary Materials). NMR data for [15]aneN_4_S are consistent with a C_2v_ symmetric structure in solution, according to the number of signals observed in the ^1^H and ^13^C-NMR spectra. Five resolved proton and carbon resonances were observed and a combination of the two-dimensional spectra HMBC, HMQC and COSY (Figures S3–S6 in Supplementary Materials), were used for the assignments. The singlet observed in the ^1^H-NMR spectrum at δ 3.34 was assigned to H*_e_*. C*_d_* at 43.65 ppm was identified through ^3^J correlation to H*_e_*, which allowed the assignment of the coupled triplets H*_d_* and H*_c_* at 3.44 and 3.57 ppm, respectively. ^3^J C-H coupling of the more shielded CH_2_ group to its symmetric counterpart, bridged by the sulphur atom, allowed the assignment of the resonances at δ 29.55 and 3.17 to C*_a_* and H*_a_*, respectively. The HMBC spectrum also exhibits three-bond CH correlation of H*_b_*, at δ 3.50, to C*_a_* and two-bond coupling to C*_d_*. The two pairs of triplets in the ^1^H-NMR spectrum display a nearly first-order A_2_X_2_ coupling pattern with some distortion. The resulting “roof effect” with a mirror image relationship is often observed for CH_2_-CH_2_ groups in an unsymmetrical environment with some strain. Leaning is more pronounced for the H*_c_*/H*_d_* pair which is closer in chemical shift (Δδ/ΔJ < 10), leading to slightly different coupling constants. For the H*_a_*/H*_b_* spin system, second order effects are negligible due to the higher chemical shift difference (Δδ/ΔJ > 20).

### 2.2. Acid-Base Behaviour

The acid-base behaviour of [15]aneN_4_S was studied by potentiometry in water at 25 °C and ionic strength 0.10 M in KNO_3_. This compound was also studied by ^1^H-NMR spectroscopy. The determined protonation constants are collected in Table 1 together with the values of the related [15]aneN_4_O and [15]aneN_5_ compounds (Figure 1) for comparison. The ligand has four basic centres. However only three constants could be accurately determined by potentiometry and the fourth one was obtained by ^1^H-NMR. The compound exhibits high and fairly high values respectively for the first two protonation constants corresponding to the protonation of nitrogen atoms in opposite positions, minimizing the electrostatic repulsion between positive charges of the ammonium groups formed. The third and fourth constants are much lower due to the stronger electrostatic repulsions as they correspond to protonation of nitrogen atoms at short distances from already protonated ones, and to the limited motion allowed in the ring backbone.

The overall basicity and all the stepwise protonation constants of [15]aneN_4_S (Table 1) are slightly lower than those of [15]aneN_4_O but even lower than those of [15]aneN_5_, as expected. Indeed sulphur and oxygen atoms have different electronic characteristics with respect to amino groups. Evidently, ethereal S and O cannot bind acidic protons, if not under particular conditions; they have different inductive effects on the adjacent aliphatic chains with respect to nitrogen and finally, they have a much lower tendency to form hydrogen bonds and are less solvated in aqueous solution than amino groups [25]. Considering these features, the introduction of S or O in the macrocyclic backbone may deeply influence the basicity of these molecules. The lower stabilization via intramolecular hydrogen bonds formation brought about by S and O in the mono and/or polyprotonated species gives rise to a consequent basicity decrease with respect to the corresponding unsubstituted polyazacycloalkanes [25].

**Table 1:** Stepwise protonation constants (log 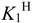) of [15]aneN_4_S and similar compounds.

Reaction equilibrium	[15]aneN_4_S ^a,b^	[15]aneN_4_O ^c^	[15]aneN_5_^d^
L + H^+^ 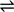 HL^+^	9.51(1)	9.66	10.01
HL^+^ + H^+^ 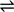 H_2_L^2+^	8.56(2)	8.77	9.28
H_2_L^2+^ + H^+^ 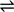 H_3_L^3+^	4.47(4)	5.30	5.87
H_3_L^3+^ + H^+^ 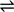 H_4_L^4+^	0.8(2) ^e^	1.2	1.84
L + 4 H^+^ 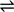 H_4_L^4+^	23.34	24.93	27.00

^a^ Values in parentheses are standard deviations on the last significant figure. ^b^ Present work *T* = 25.0 °C; *I* = 0.10 M in KNO_3_. ^c^ *T* = 25.0 °C; *I* = 0.10 M in KNO_3_; ref. [26]. ^d^ *T* = 25.0 °C; *I* = 0.10 M in KCl; ref. [27]. ^e^ Determined in this work by ^1^H-NMR spectroscopy, using the calculated value of p*K*_D4_ and the equation p*K*_D_ = 0.11 + 1.10 × p*K*_H_; ref. [28].

**Figure 2:**
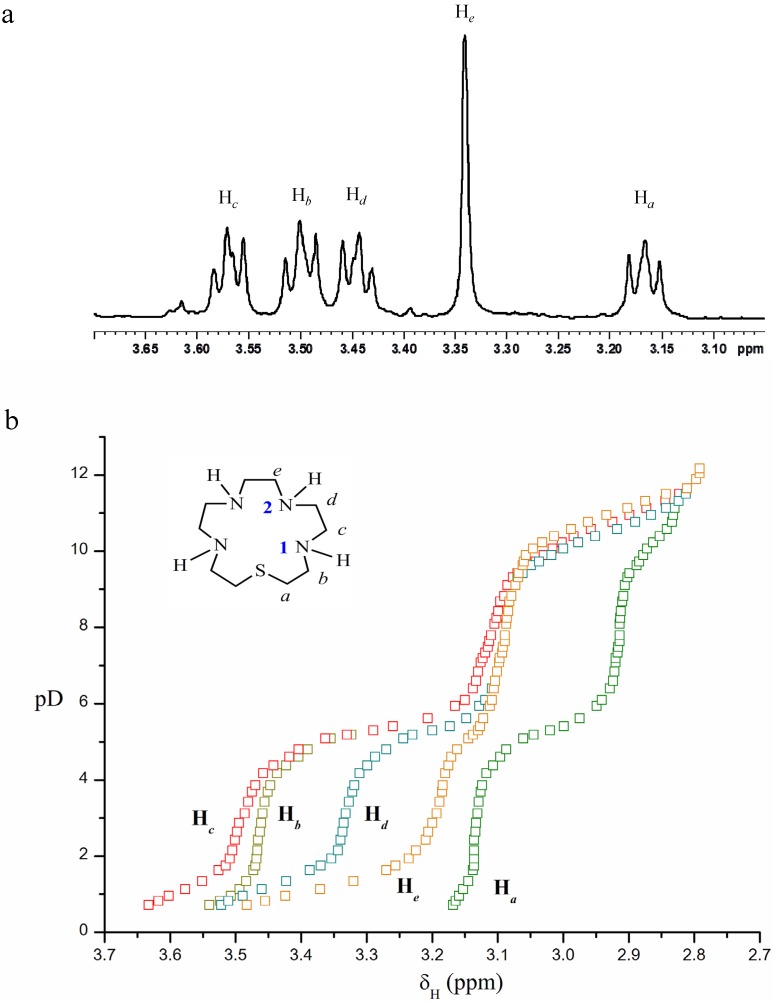
(**a**) ^1^H-NMR spectrum of [15]aneN_4_S in D_2_O, pD 1.72; (**b**) ^1^H**-**NMR titration curves for [15]aneN_4_S, chemical shift δ_H_ (ppm) in function of pD.

^1^H-NMR spectroscopic titration of [15]aneN_4_S was carried out in order to understand its protonation sequence and to determine the lower protonation constant. The ^1^H-NMR spectrum of the compound at pD 1.72 (Figure S1) and the titration curves are shown in Figure 2a,b, respectively. In the 0.72 to 4.80 pD region, five resonances were observed in the ^1^H-NMR spectra. The H*_c_* and H*_b_* resonances overlap for pD values above 5.09 until 12.17. The same behaviour was observed for the H*_e_* and H*_d_* resonances between 6.40 and 9.42. The triplets at 3.57, 3.50, 3.44 and 3.17 ppm were assigned to protons H*_c_*, H*_b_*, H*_d_* and H*_a_*, respectively, and the singlet at 3.34 ppm corresponds to H*_e_*. (Figure 2a).

The ^1^H-NMR titration curves (Figure 2b) show the effect of successive protonation of the basic centres of the molecule on the chemical shifts of the protons. The first two equivalents of acid added to the basic form of the ligand (pD 11.65–9.31) affected the four N–atoms of the compound, as all the resonances shift downfield in this pD region. The first equivalent protonates mainly N^2^–centres, since the H_e_ resonance undergoes a larger downfield shift, followed by H*_d_*, H*_c_*, H*_b_* and H*_a_*, meaning a small degree of protonation of N^1^–atoms. The second equivalent of acid protonates mainly N^1^–centres, as H*_a_*, H*_b_* and H*_c_* resonances move downfield, although simultaneous protonation of N^2^–centres is also evident, since H*_d_* and H*_e_* resonances show downfield shifts as well. 

The third equivalent of acid (pD 5.94–3.69) continues protonating the N^1^–centres, since H*_a_*, H*_b_* and H*_c_* signals show a significant shift downfield. However, a smaller downfield shift of H*_d_* and H*_e_* resonances is also observed, revealing some degree of protonation on N^2^–centres still occurring. Addition of one more equivalent of acid (pD < 2.15) causes protonation on N^2^ centres at a larger extent, according to the larger downfield shift observed for the H*_e_* and H*_d_* resonances. The last protonation occurs in a centre at a very short distance from others already protonated, and the strong repulsions then aroused in the molecule, where the motion is limited by the skeleton of the ring, renders the protonation difficult.

The ^1^H-NMR titration also allowed the determination of the protonation constants in D_2_O for [15]aneN_4_S: p*K*_D1_ = 10.90(7), p*K*_D2_ = 9.4(l), p*K*_D3_ = 5.3(1) and p*K*_D4_ = 1.0(1). These values are in agreement with the equation for the correlation between the protonation constants determined in H_2_O and in D_2_O for similar compounds: p*K*_D_ = 0.11 + 1.10 × p*K*_H_ [28].

### 2.3. Thermodynamic Stability of Metal Complexes

The stability constants of [15]aneN_4_S with Mn^2+^, Fe^2+^, Co^2+^, Ni^2+^, Cu^2+^, Zn^2+^, Cd^2+^, Hg^2+^ and Pb^2+^, determined by potentiometric titrations at 25 °C and ionic strength 0.1 M in KNO_3_, are collected in Table 2 together with the corresponding constants of complexes of the related macrocycles [15]aneN_4_O [26,29] and [15]aneN_5_ [30,31,32,33] taken from the literature for comparison. Only mononuclear ML, M(HL) and ML(OH) complexes were found. In the case of Hg^2+^ and Mn^2+^, the protonated species were not formed under our experimental conditions. For Co^2+^ and Ni^2+^, the determination of the stability constants for hydroxocomplexes was precluded since precipitation occurred (Table 2). In all cases the proposed model was accepted by the HYPERQUAD program [34] using all data points from all titration curves, with good statistical parameters. 

**Table 2:** Stepwise stability constants (log units) of the complexes of [15]aneN_4_S, [15]aneN_4_O and [15]aneN_5_ with different metal ions.

Reaction equilibrium	[15]aneN_4_S ^a,b^	[15]aneN_4_O ^c^	[15]aneN_5_^d^
Mn^2+^ + L 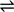 MnL^2+^	6.65(2)	8.53 ^c^	10.85 ^e^
MnL^2+^ + H^+^ 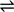 MnHL^3+^	-	-	5.04 ^e^
MnL(OH)^+^ + H^+^ 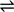 MnL^2+^	9.68(4)	-	11.22 ^e^
Fe^2+^ + L 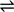 FeL^2+^	10.08(1)	10.34 ^c^	-
FeL^2+^ + H^+^ 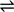 FeHL^3+^	4.83(6)	-	-
FeL(OH)^+^ + H^+^ 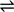 FeL^2+^	8.25(7)	pp.	-
Co^2+^ + L 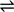 CoL^2+^	13.62(6)	12.72 ^c^	16.76 ^f^
CoL^2+^ + H^+^ 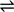 CoHL^3+^	5.20(7)	-	-
Ni^2+^ + L 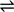 NiL^2+^	17.95(5)	14.76 ^c^	18.1 ^g^
NiL^2+^ + H^+^ 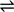 NiHL^3+^	4.00(7)	-	-
NiL(OH)^+^ + H^+ ^ 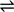 NiL^2+^	-	8.38 ^c^	-
Cu^2+^ + L 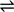 CuL^2+^	22.31(2)	20.34 ^c^	28.0 ^h^
CuL^2+^ + H^+^ 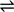 CuHL^3+^	2.49(5)	-	-
CuL(OH)^+^ + H^+ ^ 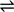 CuL^2+^	9.8(1)	10.4 ^c^	-
Zn^2+^ + L 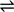 ZnL^2+^	13.472(8)	13.21 ^c^	19.1 ^i^
ZnL^2+^ + H^+^ 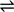 ZnHL^3+^	4.06(3)	-	3.1 ^i^
ZnL(OH)^+^ + H^+^ 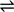 ZnL^2+^	7.16(4)	-	-
Cd^2+^ + L 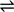 CdL^2+^	13.61(2)	13.41 ^d^	19.2 ^i^
CdL^2+^ + H^+^ 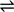 CdHL^3+^	3.97(4)	-	3.4 ^i^
CdL(OH)^+^ + H^+ ^ 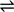 CdL^2+^	9.25(7)	-	-
Hg^2+^ + L 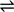 HgL^2+^	23.74(5)	-	28.5 ^j^
HgL(OH)^+^ + H^+^ 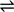 HgL^2+^	10.3(1)	-	-
Pb^2+^ + L 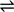 PbL^2+^	12.44(2)	12.28 ^d^	17.3 ^i^
PbL^2+^ + H^+^ 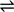 PbHL^3+^	4.44(4)	-	3.8 ^i^
PbL(OH)^+^ + H^+^ 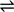 PbL^2+^	7.76(7)	-	-

^a^ Values in parentheses are standard deviations on the last significant figure. ^b^ Present work *T* = 25.0 °C; *I* = 0.10 M in KNO_3_. ^c^ *T* = 25.0 °C; *I* = 0.10 M in KNO_3_; ref. [26]. ^d^ *T* = 25.0 °C; *I* = 0.10 M in NaNO_3_; ref. [29]. ^e^ *T*= 25.0 °C; *I* = 0.10 M in NaClO_4_; ref. [30]. ^f^ *T*= 35.0 °C; *I* = 0.20 M in NaClO_4_; ref. [31]. ^g^ *T* = 35.0 °C; ref: [32]. ^h^ *T* = 25.0 °C; *I* = 0.2 M; polarographic method; ref. [35]. ^i^ *T* = 25.0 °C; *I* = 0.2 M; ref. [33]. ^j^ *T* = 25.0 °C; *I* = 0.2 M; polarographic method; ref. [33].

Potentiometric studies with [15]aneN_4_S and Ca(NO_3_)_2_ showed that both titration curves (protonation and complexation at a molar ratio 1:1) overlapped, suggesting that Ca^2+^ does not bind to the ligand. This hypothesis was supported by NMR data, as no changes were observed in the ^1^H-NMR spectrum of the ligand in presence of Ca^2+^.

**Figure 3:**
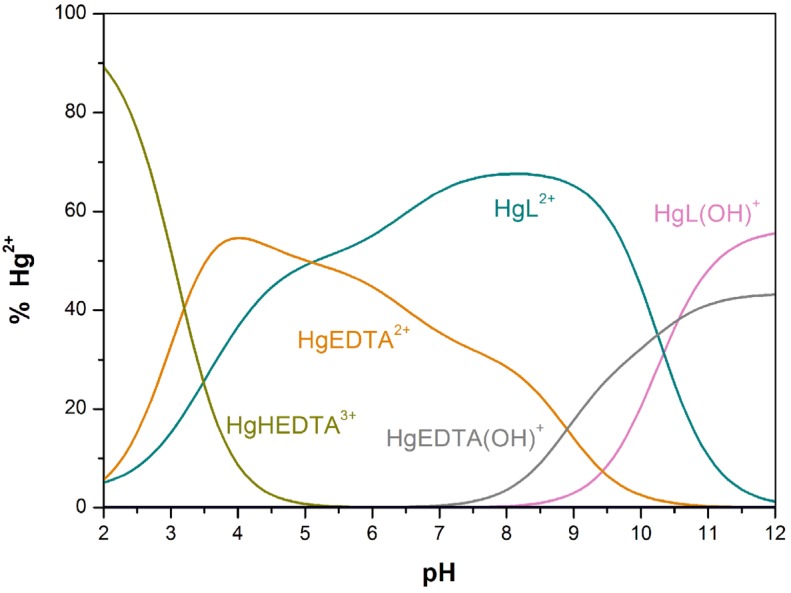
Species distribution curves calculated for an aqueous solution containing [15]aneN_4_S (L), Hg^2+^ and EDTA at 0.75:1:1 molar ratio. Percentages are given relative to Hg^2+^ at an initial value of 1.73 × 10^−3^ M.

Direct determinations of the stability constants of Cu[15]aneN_4_S^2+^ and Hg[15]aneN_4_S^2+^ were not possible as ML^2+^ was almost completely or completely formed in the beginning of the titration (pH ≈ 2.2) (Figures S9 and S11 in Supplementary Materials, respectively). Consequently, reliable values for the constants were obtained through a competition with a reference ligand, for which the protonation and stability constants are accurately known [36]. Among the various ligands tried, H_4_EDTA was chosen as the best reference ligand. Figure 3 shows the species distribution diagram of the ligand-ligand competition reaction between [15]aneN_4_S, Hg^2+^ and ethylenediaminetetraacetic acid (EDTA), obtained with the HySS program [37]. In the Supplementary Materials the species distribution curves for Cu(II) competition titration are shown (Figure S10).

[15]aneN_4_S is very selective, exhibiting a very high stability constant for Cu^2+^ (log *K*_ML_ = 22.31), and a fairly high value for Ni^2+^ (log *K*_ML_ = 17.95). On the other hand, the stability constants for the complexes of the remaining first-row transition metal ions decrease sharply. The Cd^2+^ and Pb^2+^ complexes have low stabilities. Hg^2+^ has the highest stability constant (log *K*_ML_ = 23.74) of all the metal ions studied.

In spite of the slightly lower overall basicity of [15]aneN_4_S, this ligand forms ML^2+^ complexes more stable than those of the oxatetraaza macrocycle [15]aneN_4_O, except in the case of the Mn^2+^and Fe^2+^complexes (variations of 1.88 and 0.26 log units, respectively). [15]aneN_4_S is particularly advantageous over [15]aneN_4_O for Ni^2+^ and Cu^2+^complexes, being the stability constants much higher (an increase of 3.19 and 1.97 log units, respectively, was found). Comparing with the pentaaza macrocycle [15]aneN_5_, the stability constants (log values) of the ML^2+^ metal complexes of [15]aneN_4_S are smaller except in the case of the Ni^2+^ complexes which have almost the same value (Table 2).

However, stability constants do not provide directly comparable basis for the measuring of total ion sequestering abilities of the ligands at physiological conditions (pH 7.4) and therefore they were used to calculate the pM values, defined as −log [M^2+^] (Table 3). The advantage of comparing pM values rather than stability constants is that the pM values reflect the influence of ligand basicity and metal chelate protonation. 

**Table 3:** pM values calculated for metal complexes of [15]aneN_4_S, [15]aneN_4_O and [15]aneN_5_ at pH 7.4 ^a^.

Metal ion	[15]aneN_4_S	[15]aneN_4_O	[15]aneN_5_
Mn^2+^	5.02	5.31	6.38
Fe^2+^	6.85	5.74	-
Co^2+^	10.32	9.07	12.25
Ni^2+^	14.65	11.15	13.59
Cu^2+^	19.01	16.69	23.49
Zn^2+^	10.61	9.56	14.59
Cd^2+^	10.32	9.76	14.69
Hg^2+^	20.44	-	23.99
Pb^2+^	9.3	8.63	12.79

^a^ Values calculated for 100% molar excess of the ligand over the metal ion with C_M_ = 1.0 × 10^−5^ M, based on the protonation constants and stability constants of Table 1 and Table 2, using the HySS program; ref. [37].

If the pM values of [15]aneN_4_S are compared with those of [15]aneN_4_O (Table 3) we conclude that, with the exception of Mn^2+^, all the values are higher, especially for nickel(II) and copper(II) complexes (differences, in log units, are 3.5 and 2.32, respectively). On the other hand, the comparison of pM values of the metal complexes of [15]aneN_4_S with [15]aneN_5_ (Table 3) revealed that the former complexes present lower values, except in the case of the Ni^2+^ complex, which has a higher value (difference, in log units, is 1.06). The selectivity and the high pM values for mercury(II) and copper(II) complexes of [15]aneN_4_S render this ligand of possible interest in chelation therapy.

### 2.4. Spectroscopic Studies

UV-visible-near IR spectroscopic studies of the Co(II), Ni(II) and Cu(II) complexes of [15]aneN_4_S in water solution were performed and the magnetic moments determined by the Evans method [38]. The results are collected in Table 4. 

The electronic spectrum of the pink Co[15]aneN_4_S^2+^ exhibits two principal bands, at 1075 and 504 nm, and four shoulders (Table 4), consistent with a high-spin octahedral array tetragonally distorted around the Co(II) ion [39] with one water molecule or a metal counter-ion nitrate occupying a coordination site. The octahedral field splitting parameter 10*Dq* [39] of 11450 cm^−1^ is in the expected range for relatively weak ligands. The band at 504 nm is more intense than expected for forbidden transitions in *O*_h_ complexes, which can be explained by the loss of symmetry caused by distortion. The magnetic moment of 4.9 MB is within the range for high-spin six coordinate Co(II) complexes [38]. Although five coordinate Co(II) species would also have a similar value, the absence of a weak absorption in the visible region between 830 and 670 nm, characteristic of five coordinate Co(II) complexes [39,40,41],supports the proposed six coordination for this metal ion.

**Table 4:** Spectroscopic UV-visible-near IR data and magnetic moments (µ) for the Co(II), Ni(II) and Cu(II) complexes of [15]aneN_4_S.

Complex; (colour)	pH	UV-visible-near IR ^a^ λ_max_/nm (ε, M^−1^ cm^−1^)	µ (MB)
Co[15]aneN_4_S^2+^ (pink)	6.99	1075 (4.2), 970 (sh., 4.9), 504 (67.6), 491 (sh., 99.0), 325 (sh., 2.61 × 10^3^), 270 (sh., 2.61 × 10^3^).	4.9
Ni[15]aneN_4_S^2+^ (yellow)	6.98	1040 (2.12), 945 (23.0), 847 (sh., 27.2), 813 (sh., 26.0), 528 (19.3), 310 (sh., 1.49 × 10^3^), 264 (1.91 × 10^4^).	3.1
Cu[15]aneN_4_S^2+^ (purple)	7.08	977 (23.8), 748 (sh., 16.3), 562 (45.6), 273 (1.17 × 10^3^).	1.8

^a^ sh. = shoulder.

The electronic spectrum of the yellow Ni[15]aneN_4_S^2+^ shows three absorption bands of low intensity in the visible-near IR regions and a charge transfer band at 264 nm (Table 4). 

Taking into account the work of Busch *et al.* [42], we assigned the two bands in the visible-near IR regions to transitions ^3^B_1g_ → ^3^B_2g_, (directly related to 10*Dq*^xy^) and ^3^B_1g_ → 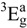 (equal to the difference between 10*Dq*^xy^ and 35/4*D*_t_). The octahedral field splitting parameter 10*Dq* and the values of the equatorial (*Dq*^xy^) and axial (*Dq*^z^) ligand field were calculated according to those assignments: 10*Dq* = 18975 cm^−1^, *Dq*^xy^ = 1898 cm^−1^ and *Dq*^z^ = 219 cm^−1^. These values, together with the ratio ν_1_/ν_2_ of 1.79 (ν_1_ and ν_2_ being the near IR and the visible absorption bands, respectively) and the magnetic moment of 3.1 MB, are characteristic of six coordinate nickel(II) environments, indicating a tetragonal (*D*_4h_) distorted octahedral geometry for this complex [43].

The purple Cu[15]aneN_4_S^2+^ complex exhibits a broad band in the visible region with the maximum centred at 565 nm assigned to the copper *d-d* transition, an intense band in the UV region and a small broad band in the near IR region (Table 4). 

**Figure 4:**
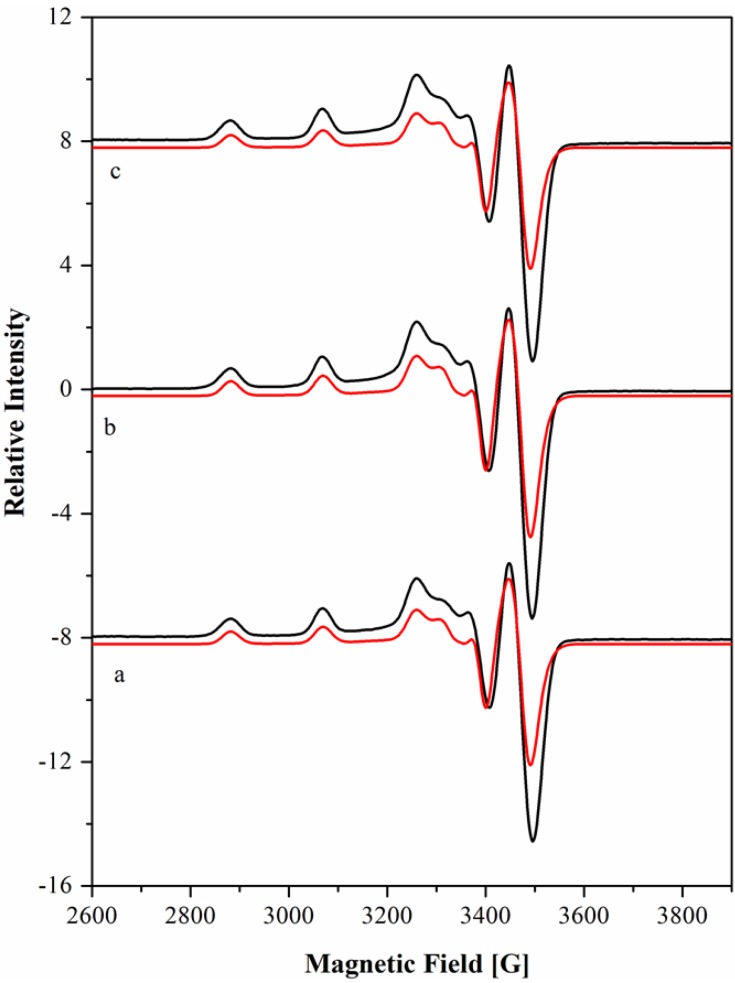
EPR X-band spectra of Cu(II) complex of [15]aneN_4_S in 1:1 ratio at pH 4.10 (**a**), 7.40 (**b**) and 9.00 (**c**) in 1.0 M of NaClO_4_. The spectra were recorded at 104 K, microwave power of 2.0 mW and modulation amplitude of 1.0 mT. The frequency (ν) was of 9.67 GHz. The simulated spectra (red lines) are below of the experimental ones (black lines).

The X-band EPR spectra of Cu[15]aneN_4_S^2+^ at the 1:1 ratio in water solution are shown in Figure 4 at three pH values. The spectra are very similar and suggest the presence of only one species. Spectroscopic visible data (λ_max_), the hyperfine coupling constants *A_i_* and *g* values, determined by the simulation of the spectra [44] (Figure 4) are compiled in Table 5 together with those of the related complex Cu[15]aneN_4_O^2+^, from the literature [45].

**Table 5:** Spectroscopic X-band EPR data for the Cu(II) complexes of [15]aneN_4_S ^a^ and the related ligand [15]aneN_4_O.

Complex	pH	Visible band λ_max_/nm (ε_molar_, M^−1^ cm^−1^)	EPR parameters (*A*_i_ × 10^4^ cm^−1^)						
			*g_x_*	*g_y_*	*g_z_*	*A_x_*	*A_y_*	*A_z_*	ref.
	9.00		2.043	2.046	2.186	30.6	28.7	192.5	-
Cu[15]aneN_4_S^2+^	7.40	565 (46)	2.042	2.045	2.182	30.3	28.0	191.1	-
	4.10		2.041	2.042	2.185	30.4	28.0	191.3	-
Cu[15]aneN_4_O^2+^	7.41	580 (141)	2.042	2.046	2.192	30.0	27.9	199.4	[45]

^a^ This work.

The parameters obtained for Cu[15]aneN_4_S^2+^ are characteristic of mononuclear copper(II) complexes in rhombic geometry with elongation of the axial bonds and a d_x2−y2_ ground state, consistent with distorted octahedral (with the sixth coordination site occupied by one water molecule or a metal counter-ion nitrate) or square pyramidal environment. Trigonal bipyramidal or tetragonal geometries involving compression of axial bonds should be excluded [46,47,48].

The hyperfine constants *A_i_* and *g* values are related to the electronic transitions by the factors derived from the ligand field theory [49,50,51]: the *g_z_* values increase and the *A_z_* values decrease when the planar ligand field becomes weaker or the axial ligand field becomes stronger, and this occurs with the simultaneous red-shift of the *d-d* absorption bands in the electronic spectra. This sequence parallels the degree of distortion from square planar to square pyramidal (*C*_4v_) and then to octahedral (*O*_h_) or tetragonal (*D*_4h_) geometries [47]. 

The EPR parameters and the electronic spectra data for the complex Cu[15]aneN_4_S^2+^ are similar to those obtained for Cu[15]aneN_4_O^2+^ (Table 5), for which a square pyramidal geometry around the copper ion was proposed, with the four nitrogen atoms in the equatorial plane and the oxygen atom occupying the apical position [45]. The Cu[15]aneN_4_S^2+^ complex presents slightly lower *g_z_* values and a small blue-shift of the absorption band, consistent with a slightly stronger equatorial ligand field. Although the coordination geometry of the complex cannot be unequivocally established without an X-ray structure, the similar results for both compounds suggest for Cu[15]aneN_4_S^2+^ a distorted square pyramidal environment around the Cu(II) centre, with the sulphur atom in the apical position and the four nitrogen atoms in the equatorial plane.

## 3. Experimental

### 3.1. General

FT-IR spectra were recorded in a Nicolet 6700 FT-IR spectrophotometer (Thermo Electron Corporation, Runcord, UK) using KBr pellets. Electrospray ionization-mass spectrometry (ESI-MS) was carried out with a Micromass Quattro Micro triple quadrupole instrument (Waters, Milford, MA, USA) and MassLynx software (version 4.1) was used for data analysis. The ionization of the compound was performed by an electrospray source in positive mode (ESI+). UV-visible spectra were recorded with a UNICAM UV-4 (Thermo Scientific, Waltham, MA, USA) and UV-visible-near IR spectra were collected with a Shimadzu UV-1603 (Tokyo, Japan). EPR spectroscopic measurements were recorded with a Bruker ESP EMX 300 spectrometer (Bruker BioSpin GmbH, Rheinstetten, Germany) equipped with continuous-flow cryostats for liquid nitrogen, operating at X-band. The compounds were characterized by ^1^H (400.13 MHz) and ^13^C-NMR (100.62 MHz) spectra recorded on a Bruker Avance 400 MHz spectrometer (Bruker BioSpin GmbH, Rheinstetten, Germany) at ≈ 20 °C probe temperature. The internal reference used for the ^1^H-NMR measurements in CDCl_3_ was tetramethylsilane (TMS) and in D_2_O was 3-(trimethylsilyl)propionic acid-*d_4_*-sodium salt (DSS). Chemical shifts (*δ*) were given in ppm and coupling constants (*J*) in Hz. Resonance assignments are based on chemical shift, peak integration and multiplicity for ^1^H-NMR spectra and on 2D experiments (COSY, HMQC and HMBC) for ^13^C-NMR spectra. FIDs were processed using the TopSpin software version 3.2 from Bruker. 

#### 3.1.1. Reagents

The dimethylester of tiodiglycolic acid (Scheme 1) was prepared according to a reported procedure [22]. Triethylenetetramine, tiodiglycolic acid and borane tetrahydrofuran complex solution 1 M in THF were purchased from Aldrich (Sigma-Aldrich, Madrid, Spain). All the commercially available chemicals were of reagent analytical grade and used as supplied without further purification. Organic solvents were purified or dried by standard methods [52].

### 3.2. Synthesis of 1-Thia-4,7,10,13-tetraazacyclopentadecane ([15]aneN_4_S)

A solution of dimethyl thiodiglycolate (3.03 g, 17 mmol) in dry methanol (100 mL) was added to a solution of triethylenetetramine (2.94 g, 20 mmol) in the same solvent (400 mL), at rt. This mixture was stirred under nitrogen at 40 °C for 9 days. The solvent was then removed under reduced pressure. The remaining oil was dissolved in a minimum amount of chloroform and purified through a silica-gel column (2.5 × 30 cm) using a mixture of CHCl_3_–MeOH (20:80 v/v) as eluent. The pure compound was dissolved in methanol and 37% hydrochloric acid was added until pH ≈ 3. The cyclic diamide precipitated as an off-yellow salt (2.94 g, 74%). IR (KBr, cm^−1^): ν 3427 (N–H), 1652 (C=O). ^1^H-NMR (400.13 MHz; D_2_O; DSS; pD = 3.4): δ 3.26 (t, 4H, (triplet), *H_d_*) 3.35 (s, 4H, (singlet), *H_a_*), 3.53 (t, 4H, *H_c_*), 3.57 (s, 4H, *H_e_*) ppm. ^13^C-NMR (100.61 MHz; D_2_O; dioxane; pD = 3.4): δ 35.3 (*C_c_*), δ 37.5 (*C_a_*), 42.8 (*C_e_*), 48.2 (*C_d_*), 175.0 (*C_2_*) ppm.

The cyclic diamide (1.10 g, 4,2 mmol) was reduced with a large excess of borane in refluxing dry THF (100 mL) under nitrogen, for eight hours. After reduction, the obtained oil was dissolved in chloroform and purified by silica-gel chromatography. The trihydrocloride salt was obtained by addition of 37% HCl until pH ≈ 2 (0.98 g, 68%). IR (KBr, cm^−1^): ν 3426 (N–H). ^1^H-NMR (400.13 MHz; D_2_O; DSS; pD = 1.72): δ 3.17 (t, 4H, ^3^*J* = 6, *H_a_*), 3.34 (s, 4H, ^3^*J* = 6, *H_e_*), 3.44 (t, 4H, *H_d_*), δ 3.50 (t, 4H, ^3^*J* = 6, *H_b_*), 3.57 (t, 4H, ^3^*J* = 6, *H_c_*) ppm. ^13^C-NMR (100.61 MHz; D_2_O; dioxane; pD = 1.72): δ 29.55 (*C_a_*), 43.64 (*C_d_*), 45.00 (*C_c_*), 45.37 (*C_e_*), 46.94 (*C_b_*). *m/z* (ESI-MS; methanol; positive ion mode) 233.20 [M + H]^+^ (Figures S7–S8 in Supplementary Materials). 

### 3.3. Potentiometric Studies

#### 3.3.1. Reagents and Solutions

All solutions were prepared with demineralized water obtained by a Millipore/Milli-Q system. Solutions of the ligand were prepared at *ca.* 2.50 × 10^−3^ M and their exact concentrations were obtained by titration with the standardised solution of KOH.

Metal ion solutions were prepared at about 0.050 M from nitrate salts (analytical grade), except in the case of mercury nitrate (*ca*. 0.01 M) that was kept in excess of nitric acid to prevent precipitation. The solutions were standardised by titration with Na_2_H_2_EDTA [53]. 

Carbonate-free solutions of the titrant KOH were freshly prepared by dilution of a commercial ampoule of Fixanal (Fluka, Sigma-Aldrich, Madrid, Spain) at *ca*. 0.10 M, under a stream of pure argon gas. Solutions were discarded every time carbonate concentration was about 0.5% of the total amount of base. A 0.100 M standard solution of HNO_3_ (prepared from a Merck ampoule) was used for the back titrations. The titrant solutions were standardised (tested by Gran's method) [54]. For the competition titrations a standard K_2_H_2_EDTA aqueous solution was used.

The equipment used has been described before [7]. The temperature was controlled at 25.0 ± 0.1 °C. CO_2_ was excluded from the titration cell during experiments by passing argon across the experimental solution. The ionic strength of the solutions was kept at 0.10 ± 0.01 M with KNO_3_.

#### 3.3.2. Measurements

The [H^+^] of the solutions was determined by the measurement of the electromotive force (emf) of the cell, *E* = *E'^o^* + *Q* log[H^+^] + *E*_j_. *E'^o^* and *Q* were determined by titration of a solution of known hydrogen-ion concentration at the same ionic strength, using the acid pH range of the titration. *E*_j _was found to be negligible under our experimental conditions. The value of *K_w_* was obtained from data acquired in the alkaline range of the titration, considering *E'^o^* and *Q* valid for the entire pH range and found to be equal to 10^−13.80^ M^2^ in our experimental conditions. The electromotive force data were determined after additions of 0.050 mL increments of standardised KOH solution, and after stabilization in this direction, equilibrium was then approached from the other direction by adding standard 0.100 M nitric acid (back titration). Before and after each determination, a calibration of the system was performed by titration of a 2.00 × 10^−3^ M HNO_3_ solution. 

The potentiometric equilibrium measurements were carried out using 20.00 mL of *≈* 2.50 × 10^−3^ M ligand solutions diluted to a final volume of 30.00 mL, in the absence of metal ions and in the presence of each metal ion for which the C_M_:C_L_ ratio was 1:1. A minimum of two replicate measurements was made. For the reactions of Cu^2+^ and Hg^2+^, ligand-ligand competition titrations were performed to determine the stability constants. K_2_H_2_EDTA was used as the second ligand in ratios C_L_^1^: C_L_: C_M_ 1:0.75:1, being L^1^, K_2_H_2_EDTA [55]. EDTA protonation and copper(II) stability constants values were determined before, under the same experimental conditions: log *K*_1_^H^ = 10.22, log *K*_2_^H^ = 6.16, log *K*_3_^H^ = 2.71, log *K*_4_^H^ = 2.0, log *K*_CuEDTA_ = 19.23, log *K*_CuHEDTA_ = 3.06, log *K*_CuEDTAOH_ = 11.33 [55]; for the Hg(II) complexes the following values were selected from the literature: log *K*_HgEDTA_ = 21.50, log *K*_HgHEDTA_ = 3.20, log *K*_HgEDTAOH_ = 8.90 [56]. 

#### 3.3.3. Calculation of Equilibrium Constants

Overall equilibrium constants 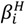 and 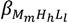 (being 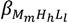 = [M*_m_*H*_h_*L*_l_*]/[M]*^m^* [H]*^h^* [L]*^l^*) were calculated by fitting the potentiometric data from protonation or complexation titrations with the HYPERQUAD program [34]. Species distribution diagrams were plotted from the calculated constants with the HySS program [37]. Only mononuclear species, ML, MHL and MH_-1_L were found for the metal complexes of [15]aneN_4_S (being *β*_MH-1L_ = *β*_MLOH_ × *K*_w_). The hydrolysis constants of the metal ions were taken from literature and kept constant for the calculations. Differences, in log units, between the values of *β*_MHL_ (or *β*_MH-1L_) and *β*_ML_ constants, provide the stepwise reaction constants. The species considered in a particular model were those that could be justified by the principles of coordination chemistry. The errors quoted are the standard deviations of the overall stability constants given directly by the program for the input data, which include all the experimental points of all titration curves, and were determined by the normal propagation rules for the stepwise constants.

### 3.4. Spectroscopic Studies

^1^H-NMR titration was performed with a 4.80 × 10^−2^ M solution of [15]aneN_4_S prepared in D_2_O. The pD values were adjusted by adding DCl or CO_2_-free KOD solutions. Following each addition, the −log [H^*^] was measured after equilibration, directly in the NMR tube with an Orion 3 Star pH meter equipped with a combined glass Ag-AgCl microelectrode U402-M3-S7/200, Mettler-Toledo (Barcelona, Spain). 

The electrode was previously calibrated with standard aqueous buffers solutions and the pD values were calculated according to the equation pD = pH^*^ + (0.40 ± 0.02), where pH^*^ corresponds to the reading of the pH meter [28].

The dissociation constants in D_2_O (p*K*_D_) were determined from the ^1^H-NMR titration by using the HypNMR program [57]. These p*K*_D_ values were converted to p*K*^H^ values obtained in water by the equation p*K*_D_ = 0.11 + 1.10 × p*K*_H_ [28].

Magnetic moments were measured at 293.6 K using aqueous solutions of Co[15]aneN_4_S^2+^ (1.99 × 10^−3^ M, pH 7.02), Ni[15]aneN_4_S^2+^ (2.39 × 10^−3^ M, pH 7.11) and Cu[15]aneN_4_S^2+^ (3.98 × 10^−3^ M, pH 7.09). The ^1^H-NMR spectra of the solutions with DSS as internal reference, were acquired in a tube containing an internal capillary filled with D_2_O and DSS, and the corresponding magnetic moments calculated from the shift (Δδ) between both reference signals, according to Evans method [38].

Electronic spectra were recorded using aqueous solutions of Co^2+^, Ni^2+^ and Cu^2+^ complexes for visible-near IR regions of 1.21 × 10^−2^ M, 1.23 × 10^−2^ M and 1.29 × 10^−2^ M at pHs 6.99, 6.98, 7.08, respectively; for UV region 1.02 × 10^−3^ M, 2.04 × 10^−3^ M and 2.02 × 10^−3^ M at pHs 6.92, 6.87 and 7.08, respectively.

EPR spectroscopy measurements of copper(II) complexes of [15]aneN_4_S were performed at 104 K. The complexes were prepared in 1.25 × 10^−3^ M, at pH 4.10, 7.40 and 9.00, in 1 M NaClO_4_ aqueous solutions. 

## 4. Conclusions

A thiatetraaza macrocycle [15]aneN_4_S having four nitrogen and one sulphur as donor atoms has been synthesised. Potentiometric studies have shown that this compound has a high selectivity towards Hg(II) and Cu(II) over the other divalent metal ions under study. The UV-visible-near IR spectroscopies and magnetic moment data for the Co(II) and Ni(II) complexes indicated a tetragonal distorted coordination geometry for both metal centres. The value of magnetic moment and the X-band EPR spectra of the Cu(II) complex are consistent with a distorted square pyramidal geometry.

This work suggest that [15]aneN_4_S should be evaluated as a potential ligand to be used in chelation therapy on disorders triggered by these metal ions. Further studies would be performed in order to evaluate the efficacy of this chelator in the biological milieu.

## Supplementary Materials

Supplementary materials can be acceded at: http://www.mdpi.com/1420-3049/19/1/550/s1.

## References

[1] Hayes R.B. (1997). The carcinogenicity of metals in humans. Cancer Cause. Control.

[2] Järup L. (2003). Hazards of heavy metal contamination. Br. Med. Bull..

[3] Caussy D., Gochfeld M., Gurzau E., Neagu C., Ruedel H. (2003). Lessons from case studies of metals: Investigating exposure, Bioavailability, and risk. Ecotoxicol. Environ. Saf..

[4] Blanuša M., Varnai V.M., Piasek M., Kostial K. (2005). Chelators as antidotes of metal toxicity: Therapeutic and experimental aspects. Curr. Med. Chem..

[5] Flora S.J.S., Pachauri V. (2010). Chelation in metal intoxication. Int. J. Environ. Res. Public Health.

[6] Sears M.E. (2013). Chelation: Harnessing and enhancing heavy metal detoxification—A review. Sci. World J..

[7] Fernandes A.S., Cabral M.F., Costa J., Castro M., Delgado R., Drew M.G., Felix V. (2011). Two macrocyclic pentaaza compounds containing pyridine evaluated as novel chelating agents in copper(II) and nickel(II) overload. J. Inorg. Biochem..

[8] Andersen O., Aaseth J. (2002). Molecular mechanisms of *in vivo* metal chelation: Implications for clinical treatment of metal intoxications. Environ. Health. Perspect..

[9] Andersen O. (2004). Chemical and biological considerations in the treatment of metal intoxications by chelating agents. Mini Rev. Med. Chem..

[10] Aposhian H.V., Maiorino R.M., Gonzalez-Ramirez D., Zuniga-Charles M., Xu Z., Hurlbut K.M., Junco-Munoz P., Dart R.C., Aposhian M.M. (1995). Mobilization of heavy metals by newer, Therapeutically useful chelating agents. Toxicology.

[11] Gonçalves S., Fernandes A.S., Oliveira N.G., Marques J., Costa J., Cabral M.F., Miranda J., Cipriano M., Guerreiro P.S., Castro M. (2012). Cytotoxic effects of cadmium in mammary epithelial cells: Protective role of the macrocycle [15]pyN5. Food Chem. Toxicol..

[12] Mewis R.E., Archibald S. (2010). Biomedical applications of macrocyclic ligand complexes. Coord. Chem. Rev..

[13] Hancock R.D., Martell A.E. (1989). Ligand design for selective complexation of metal ions in aqueous solution. Chem. Rev..

[14] Hancock R.D., Dobson S.M., Boeyens J.C.A. (1987). Metal ion size selectivity of 1-Thia-4, 7-diazacyclononane (9-aneN_2_S), and other tridentate macrocycles. A study by molecular mechanics calculation, structure determination, and formation constant determination of complexes of 9-aneN_2_S. Inorganica Chim. Acta.

[15] Westerby B.C., Juntunen K.L., Leggett G.H., Pett V.B., Koenigbauer M.J., Purgett M.D., Taschner M.J., Ochrymowycz L.A., Rorabacher D.B. (1991). Macrocyclic polyamino polythiaether ligands with N_x_S_4-x_ and N_x_S_5-x_ donor sets: Protonation constants, stability constants, and kinetics of complex formation with the aquocopper(II) ion. Inorg. Chem..

[16] Arnaud-Neu F., Schwing-Weill M.J., Louis R., Weiss R. (1979). Thermodynamic and spectroscopic properties in aqueous solutions of pentadentate macrocyclic complexes. Inorg. Chem..

[17] Balakrishnan K.P., Kaden T.A., Siegfried L., Zuberbühler A.D. (1984). Stabilities and redox properties of Cu(I) and Cu(II) complexes with macrocyclic ligands containing the N_2_S_2_ donor set. Helv. Chim. Acta.

[18] Walker T.L., Malasi W., Bhide S., Parker T., Zhang D., Freedman A., Modarelli J.M., Engle J.T., Ziegler C.J., Custer P. (2012). Synthesis and characterization of 1,8-dithia-4,11-diazacyclotetradecane. Tetrahedron Lett..

[19] Papini G., Alidori S., Lewis J.S., Reichert D.E., Pellei M., Lobbia G.G., Biddlecombe G.B., Anderson C.J., Santini C. (2009). Synthesis and characterization of the copper(II) complexes of new N_2_S_2_-donor macrocyclic ligands: Synthesis and *in vivo* evaluation of the ^64^Cu complexes. Dalton Trans..

[20] Aquilanti G., Giorgetti M., Minicucci M., Papini G., Pellei M., Tegoni M., Trasatti A., Santini C. (2011). A study on the coordinative versatility of new N,S-donor macrocyclic ligands: XAFS, and Cu^2+^ complexation thermodynamics in solution. Dalton Trans..

[21] Kodama M., Koike T., Hoshiga N., Machida R., Kimura E. (1984). Metal chelates of sulphur-containing polyamine macrocycles and oxygenation of the corresponding cobalt(II) complexes. J. Chem. Soc. Dalton Trans..

[22] Vollhardt K.P.C., Schore N.E. (2011). Organic Chemistry.

[23] Tabushi I., Okino H., Kuroda Y. (1976). Convenient synthesis of macrocyclic-compounds containing two of nitrogen, Oxygen or sulphur atoms. Tetrahedron Lett..

[24] Steenland M.W.A., Westbroek P., Dierck I., Herman G.G., Lippens W., Temmerman E., Goeminne A.M. (1999). Aqueous solution study of Cu(II) and Ni(II) complexes of macrocyclic oxa- and thia- containing trans-dioxo-tetraamines. Polyhedron.

[25] Bencini A., Bianchi A., Garcia-España E., Micheloni M., Ramirez J.A. (1999). Proton coordination by polyamine compounds in aqueous solution. Coord. Chem. Rev..

[26] Cabral M.F., Delgado R. (1994). Metal complexes of pentadentate macrocyclic ligands containing oxygen and nitrogen as donor atoms. Helv. Chim. Acta.

[27] Motekaitis R.J., Rogers B.E., Reichert D.E., Martell A.E., Welch M.J. (1996). Stability and structure of activated macrocycles. Ligands with biological applications. Inorg. Chem..

[28] Delgado R., Fraústo da Silva J.J.R., Amorim M.T.S., Cabral M.F., Chaves S., Costa J. (1991). Dissociation constants of Brønsted acids in D_2_O and H_2_O: Studies on polyaza and polyoxa-polyaza macrocycles and a general correlation. Anal. Chim. Acta.

[29] Hancock R.D., Wade P.W., Ngwenya M.P., de Sousa A.S., Damu K.V. (1990). Ligand design for complexation in aqueous solution. 2. Chelate ring size as a basis for control of size-based selectivity for metal ions. Inorg. Chem..

[30] Riley D.P., Henke S.L., Lennon P.J., Weiss R.H., Neumann W.L., Rivers W.J., Aston K.W., Sample K.R., Rahman H., Ling C.S. (1996). Synthesis, characterization, and stability of manganese(II) C-substituted 1,4,7,10,13-pentaazacyclopentadecane complexes exhibiting superoxide dismutase activity. Inorg. Chem..

[31] Kodama M., Kimura E. (1980). Effects of axial ligation on molecular oxygen binding by donor atoms built in saturated macrocycles. Equilibrium and kinetic study with cobalt(II) complexes of macrocyclic pentaamines and oxatetraamine. Inorg. Chem..

[32] Kodama M., Kimura E., Yamaguchi S. (1980). Complexation of the macrocyclic hexa-amine ligand 1,4,7,10,13,16-hexa-azacyclo-octadecane(“18-azacrown-6”). J. Chem. Soc. Dalton Trans..

[33] Kodama M., Kimura E. (1978). Equilibria of complex formation between several bivalent metal ions and macrocyclic tri- and penta-amines. J. Chem. Soc. Dalton Trans..

[34] Gans P., Sabatini A., Vacca A. (1996). Investigation of equilibria in solution. Determination of equilibrium constants with the HYPERQUAD suite of programs. Talanta.

[35] Kodama M., Kimura E. (1978). Effects of cyclization and ring size on complex formation between penta-amine ligands and copper(II). J. Chem. Soc. Dalton Trans..

[36] Costa J., Delgado R., Drew M.G.B., Félix V. (1998). Design of selective macrocyclic ligands for the divalent first-row transition-metal ions. J. Chem. Soc. Dalton Trans..

[37] Alderighi L., Gans P., Ienco A., Peters D., Sabatini A., Vacca A. (1999). Hyperquad simulation and speciation (HySS): A utility program for the investigation of equilibria involving soluble and partially soluble species. Coord. Chem. Rev..

[38] Evans D.F. (1959). The determination of the paramagnetic susceptibility of substances in solution by nuclear magnetic resonance. J. Chem. Soc..

[39] Lever A.B.P. (1984). Inorganic Electronic Spectroscopy.

[40] Banci L., Bencini A., Benelli C., Gatteschi D., Zanchini C. (1982). Spectral-structural correlations in high-spin cobalt(II) complexes. Sruct. Bond..

[41] Bertini I., Luchinat C. (1984). High spin cobalt(II) as a probe for the investigation of metalloproteins. Adv. Inorg. Biochem..

[42] Martin L.Y., Sperati C.R., Busch D.H. (1977). The spectrochemical properties of tetragonal complexes of high spin nickel(II) containing macrocyclic ligands. J. Am. Chem. Soc..

[43] Sacconi L., Mani F., Bencini A., Wilkinson G., Gillard R.D., McCleverty J.A. (1987). Nickel. Comprehensive Coordination Chemistry.

[44] Neese F. (1993). Electronic Structure and Spectroscopy of Novel Copper Chromophores in Biology. Diploma Thesis.

[45] Fernandes A.S., Gaspar J., Cabral M.F., Caneiras C., Guedes R., Rueff J., Castro M., Costa J., Oliveira N.G. (2007). Macrocyclic copper(II) complexes: Superoxide scavenging activity, Structural studies and cytotoxicity evaluation. J. Inorg. Biochem..

[46] Li Y. (1996). X-Ray structures and spectroscopic studies of diaqua- and dichlorocopper(II) complexes of 15 crown 5 and 4' substituted benzo-15-Crown-5 with a 3dx(x=z2) ground state doublet. Bull. Chem. Soc. Jpn..

[47] Barbaro P., Bianchini C., Capannesi G., di Luca L., Laschi F., Petroni D., Salvadori P.A., Vacca A., Vizza F. (2000). Synthesis and characterization of the tetraazamacrocycle 4,10-dimethyl-1,4,7,10-tetraazacyclododecane-1,7-diacetic acid (H_2_Me_2_DO2A) and of its neutral copper(II) complex [Cu(Me_2_DO2A)]. A new ^64^Cu-labeled macrocyclic complex for positron emission tomography imaging. J. Chem. Soc. Dalton Trans..

[48] Hathaway B.J. (1983). Copper. Coord. Chem. Rev..

[49] Gersmann H.R., Swalen J.D. (1962). Electron paramagnetic resonance spectra of copper complexes. J. Chem. Phys..

[50] Yokoi H., Sai M., Isobe T., Ohsawa S. (1972). ESR studies of the copper(II) complexes of amino acids. Bull. Chem. Soc. Jpn..

[51] Lau P.W., Lin W.C. (1975). Electron spin resonance and electronic structure of some metalloporphyrins. J. Inorg. Nucl. Chem..

[52] Perrin D.D., Armarego W.L.F. (1988). Purification of Laboratory Chemicals.

[53] Schwarzenbach G., Flaschka W. (1969). Complexometric Titrations.

[54] Rossotti F.J.C., Rossotti H. (1965). Potentiometric titrations using Gran plots: A textbook omission. J. Chem. Educ..

[55] Delgado R., do Carmo Figueira M., Quintino S. (1997). Redox method for the determination of stability constants of some trivalent metal complexes. Talanta.

[56] Pettit L.D., Powell H.K.J. (2003). IUPAC Stability Constants Database.

[57] Frassineti C., Ghelli S., Gans P., Sabatini A., Moruzzi M.S., Vacca A. (1995). Nuclear magnetic resonance as a tool for determining protonation constants of natural polyprotic bases in solution. Anal. Biochem..

