# Supplementary material for: [15]aneN4S: Synthesis, Thermodynamic Studies and Potential Applications in Chelation Therapy
Source: Molecules. 2014 Jan 3;19(1):550–67. doi: 10.3390/molecules19010550 (PMC6271953; doi:10.3390/molecules19010550)

Supplementary Materials for
[15]aneN_4S: Synthesis, Thermodynamic Studies and Potential Applications in Chelation Therapy

	Contents	Page
**Table S1**	Assignment of ^{1}H- and ^{13}C-NMR data for [15]aneN_4S in D_2O at pD 1.72.	S1
**Figure S1**	^{1}H-NMR spectrum of [15]aneN_4S in D_2O at pD 1.72.	S1
**Figure S2**	^{13}C-NMR spectrum of [15]aneN_4S in D_2O at pD 1.72.	S2
**Figure S3**	^{1}H-^{13}C HMBC of [15]aneN_4S in D_2O at pD 1.72.	S2
**Figure S4**	^{1}H-^{13}C HMQC of [15]aneN_4S in D_2O at pD 1.72.	S2
**Figure S5**	^{1}H-^{1}H COSY of [15]aneN_4S in D_2O at pD 1.72.	S3
**Figure S6**	COSY (↔) and HMBC (→) correlations for [15]aneN_4S, in D_2O, pD 1.72.	S3
**Figure S7**	MS Scan of [15]aneN_4S by direct infusion.	S3
**Figure S8**	Theoretical and Experimental MS Scans of [15]aneN_4S by direct infusion.	S4
**Figure S9**	Species distribution curves calculated for an aqueous solution containing [15]aneN_4S (L) and Cu^{2+} at 1:1 molar ratio. Percentages are given relative to Cu^{2+} at an initial value of 1.71×10^{-3} M.	S4
**Figure S10**	Species distribution curves calculated for an aqueous solution containing [15]aneN_4S (L), Cu^{2+} and EDTA at 0.75:1:1 molar ratio. Percentages are given relative to Cu^{2+} at an initial value of 1.71×10^{-3} M.	S5
**Figure S11**	Species distribution curves calculated for an aqueous solution containing [15]aneN_4S (L) and Hg^{2+} at 1:1 molar ratio. Percentages are given relative to Hg^{2+} at an initial value of 1.73×10^{-3} M.	S5

Table S1. Assignment of ^{1}H- and ^{13}C-NMR data for [15]aneN_4S in D_2O at pD 1.72.

C/H labels	^{1}H δ (ppm)	*J* (Hz)	^{13}C δ (ppm)
a	3.17 (4 H, t)	6.0	29.55
b	3.50 (4 H, t)	4.0, 5.2	46.94
c	3.57 (4 H, t)	4.0, 5.2	45.00
d	3.44 (4 H, t)	6.0	43.65
e	3.34 (4 H, s)	-	45.38

Figure S1. ^{1}H-NMR spectrum of [15]aneN_4S in D_2O at pD 1.72.

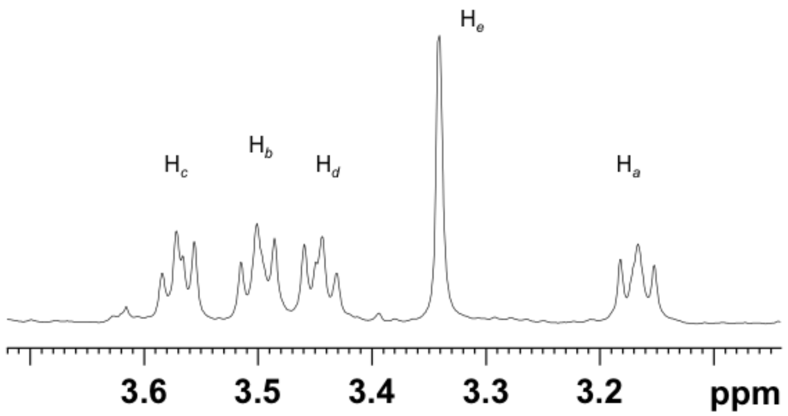

Figure S2. ^{13}C-NMR spectrum of [15]aneN$_4$S in D_2O at pD 1.72.

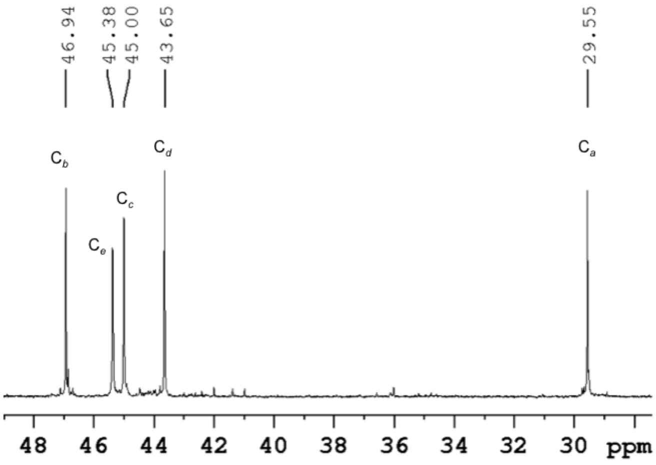

Figure S3. ^{1}H-^{13}C HMBC of [15]aneN$_4$S in D_2O at pD 1.72.

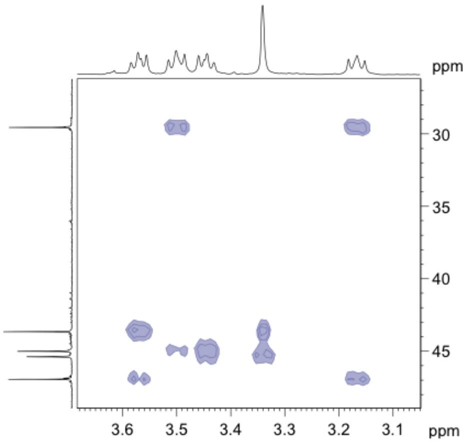

Figure S4. ^{1}H-^{13}C HMQC of [15]aneN$_4$S in D_2O at pD 1.72.

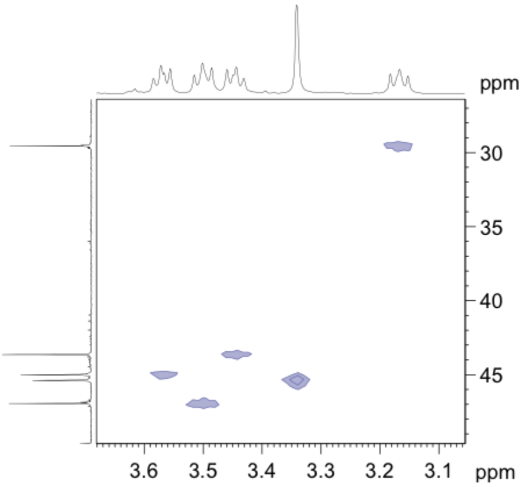

Figure S5. ^1H-^1H COSY of [15]aneN_4S in D_2O at pD 1.72.

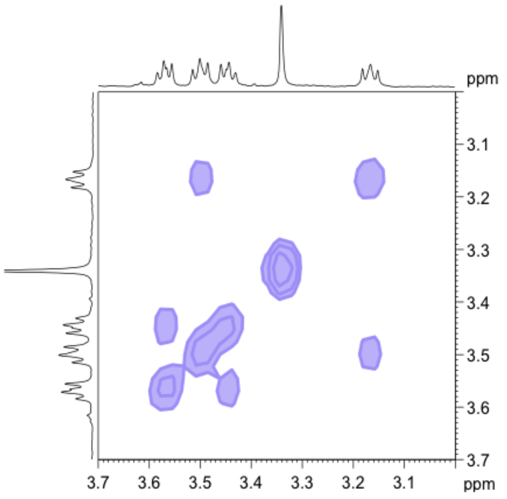

Figure S6. COSY (↔) and HMBC (→) correlations for [15]aneN_4S, in D_2O, pD 1.72.

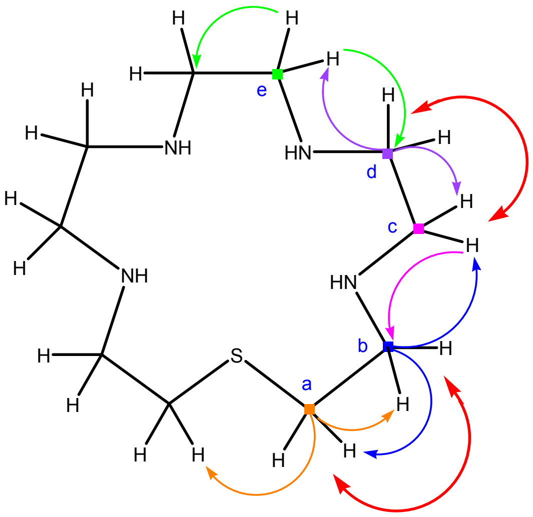

Figure S7. MS Scan of [15]aneN_4S by direct infusion.

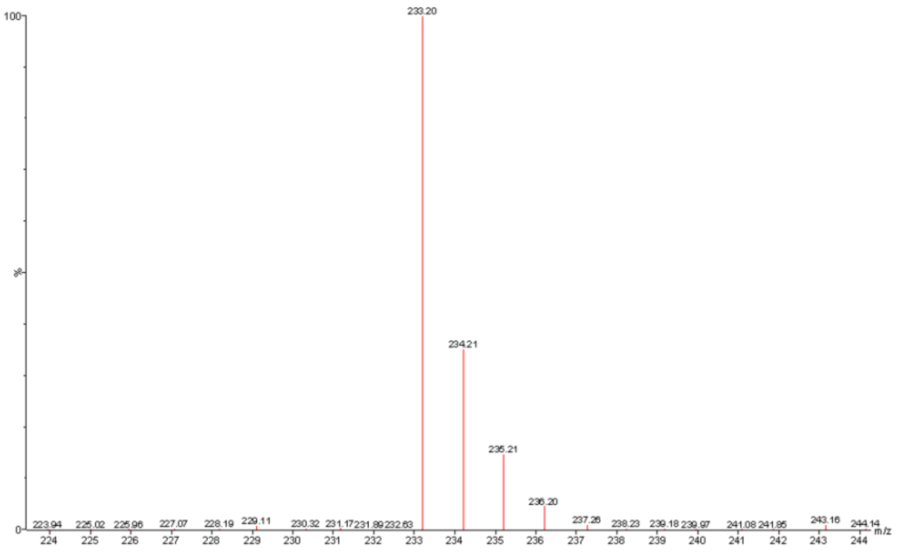

Figure S8. Theoretical and Experimental MS Scans of [15]aneN_4S by direct infusion.

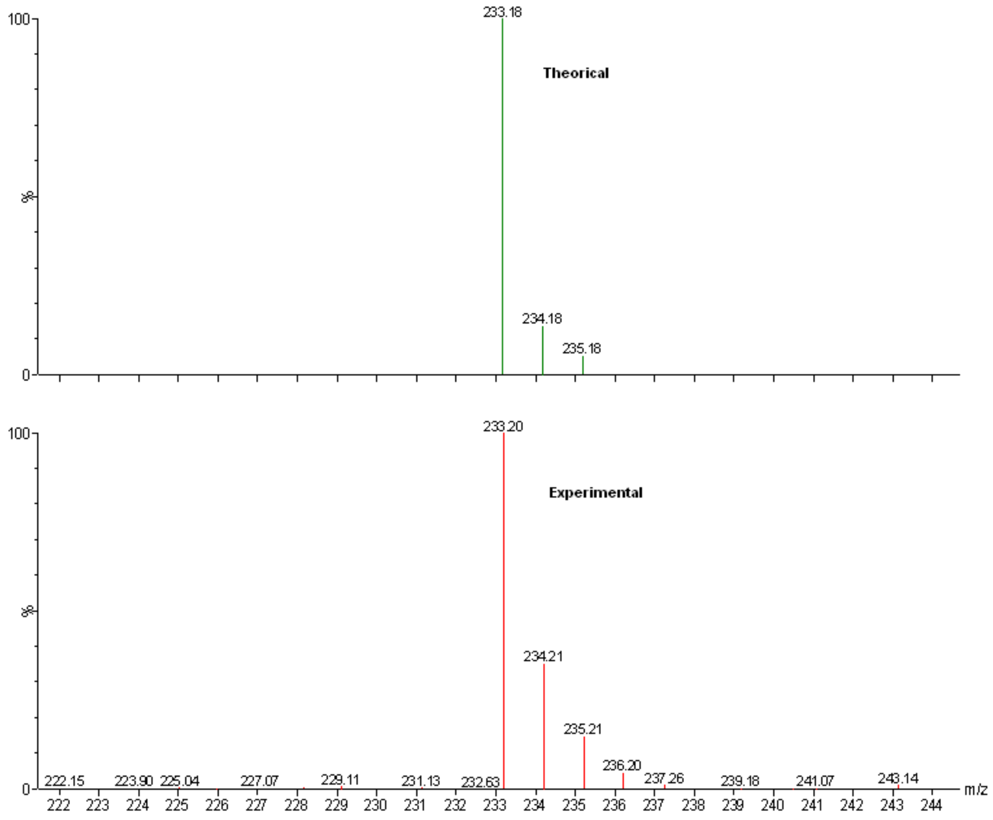

Figure S9. Species distribution curves calculated for an aqueous solution containing [15]aneN_4S (L) and Cu^{2+} at 1:1 molar ratio. Percentages are given relative to Cu^{2+} at an initial value of 1.71×10^{-3} M.

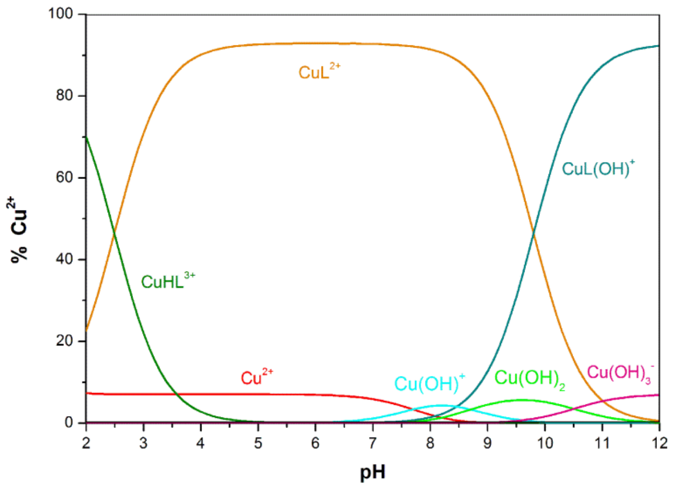

Figure S10. Species distribution curves calculated for an aqueous solution containing [15]aneN$_4$S (L), Cu^{2+} and EDTA at 0.75:1:1 molar ratio. Percentages are given relative to Cu^{2+} at an initial value of 1.71 × 10^{-3} M.

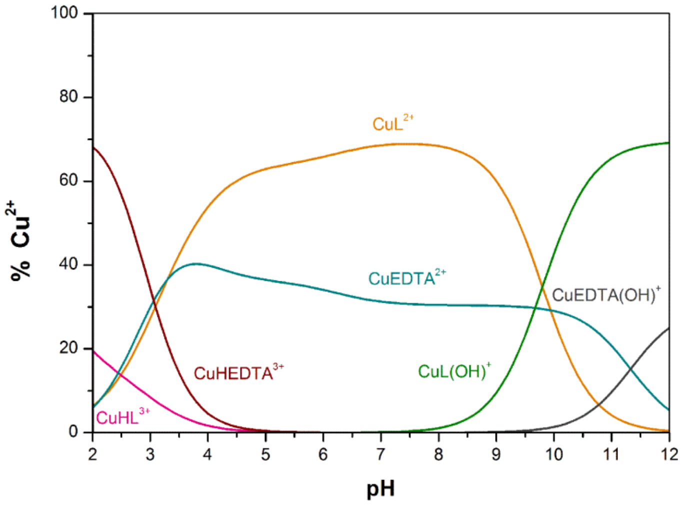

Figure S11. Species distribution curves calculated for an aqueous solution containing [15]aneN$_4$S (L) and Hg^{2+} at 1:1 molar ratio. Percentages are given relative to Hg^{2+} at an initial value of 1.73 × 10^{-3} M.

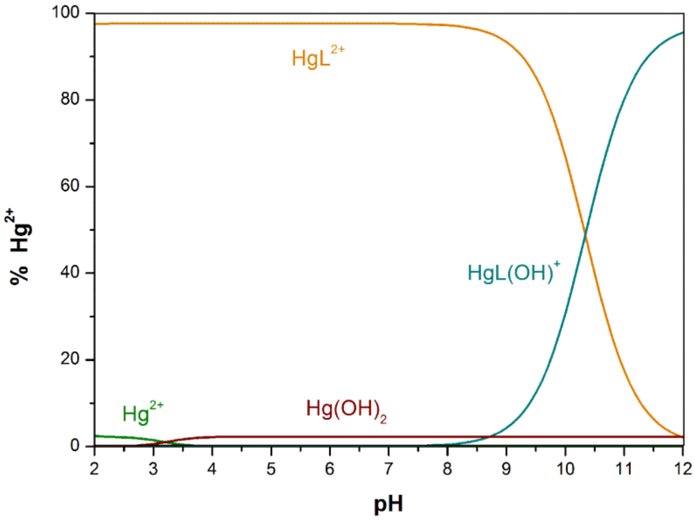

Supplement: Supplementary file 1 [file molecules-19-00550-s001.pdf]
