# Supplementary material for: [15]aneN4S: Synthesis, Thermodynamic Studies and Potential Applications in Chelation Therapy
Source: Molecules. 2014 Jan 3;19(1):550–67. doi: 10.3390/molecules19010550 (PMC6271953; doi:10.3390/molecules19010550)
Supplement: Supplementary file 2 [file molecules-19-00550-s002.pdf]

Correction of Acknowledgments

In the original published version of this paper we did not correctly and fully acknowledge our financial support. The acknowledgment is hereby additionally published as follows.
This work is financed by National funds through FCT – Fundação para a Ciência e Tecnologia under the project Ref. FCT PEst-OE/SAU/UI4013/2014.
